# The Role of Vitamin D in Modulating Mesenchymal Stem Cells and Endothelial Progenitor Cells for Vascular Calcification

**DOI:** 10.3390/ijms21072466

**Published:** 2020-04-02

**Authors:** Yi-Chou Hou, Chien-Lin Lu, Cai-Mei Zheng, Wen-Chih Liu, Tzung-Hai Yen, Ruei-Ming Chen, Yuh-Feng Lin, Chia-Ter Chao, Kuo-Cheng Lu

**Affiliations:** 1 Division of Nephrology, Department of Medicine, Cardinal-Tien Hospital, New Taipei City 231, Taiwan; athletics910@gmail.com; 2 School of Medicine, Fu-Jen Catholic University, New Taipei City 234, Taiwan; janlin0123@gmail.com; 3 Graduate Institute of Clinical Medicine, College of Medicine, Taipei Medical University, Taipei 110, Taiwan; 11044@s.tmu.edu.tw (C.-M.Z.); wayneliu55@gmail.com (W.-C.L.); linyf@shh.org.tw (Y.-F.L.); 4 Division of Nephrology, Department of Medicine, Fu-Jen Catholic University Hospital, New Taipei City 243, Taiwan; 5 Division of Nephrology, Department of Internal Medicine, School of Medicine, College of Medicine, Taipei Medical University, Taipei 110, Taiwan; 6 Division of Nephrology, Department of Internal Medicine, Shuang Ho Hospital, Taipei Medical University, Taipei 235, Taiwan; 7 Division of Nephrology, Department of Internal Medicine, Tungs’ Taichung Metroharbor Hospital, Taichung City 43304, Taiwan; 8 Department of Nephrology, Chang Gung Memorial Hospital, Taoyuan 333, Taiwan; m19570@adm.cgmh.org.tw; 9 College of Medicine, Chang Gung University, Taoyuan 333, Taiwan; 10 Graduate Institute of Medical Sciences, College of Medicine, Taipei Medical University, Taipei 110, Taiwan; rmchen@tmu.edu.tw; 11 Graduate Institute of Toxicology, National Taiwan University College of Medicine, Taipei 104, Taiwan; 12 Nephrology division, Department of Internal Medicine, National Taiwan University Hospital, Taipei 100, Taiwan; 13 Department of Internal Medicine, National Taiwan University Hospital BeiHu Branch, Taipei 108, Taiwan; 14 Division of Nephrology, Taipei Tzu Chi Hospital, Buddhist Tzu Chi Medical Foundation, and School of Medicine, Buddhist Tzu Chi University, Hualien, Taiwan

**Keywords:** vascular calcification, vitamin D, mesenchymal stem cell, endothelial progenitor cell

## Abstract

Vascular calcification, which involves the deposition of calcifying particles within the arterial wall, is mediated by atherosclerosis, vascular smooth muscle cell osteoblastic changes, adventitial mesenchymal stem cell osteoblastic differentiation, and insufficiency of the calcification inhibitors. Recent observations implied a role for mesenchymal stem cells and endothelial progenitor cells in vascular calcification. Mesenchymal stem cells reside in the bone marrow and the adventitial layer of arteries. Endothelial progenitor cells that originate from the bone marrow are an important mechanism for repairing injured endothelial cells. Mesenchymal stem cells may differentiate osteogenically by inflammation or by specific stimuli, which can activate calcification. However, the bioactive substances secreted from mesenchymal stem cells have been shown to mitigate vascular calcification by suppressing inflammation, bone morphogenetic protein 2, and the Wingless-INT signal. Vitamin D deficiency may contribute to vascular calcification. Vitamin D supplement has been used to modulate the osteoblastic differentiation of mesenchymal stem cells and to lessen vascular injury by stimulating adhesion and migration of endothelial progenitor cells. This narrative review clarifies the role of mesenchymal stem cells and the possible role of vitamin D in the mechanisms of vascular calcification.

## 1. Introduction

Vascular calcification, which involves the deposition of calcifying particles within the endothelial layer or smooth muscle within the medial layer, is an important issue due to its associated complications, such as peripheral arterial occlusive disease and coronary artery disease [1,2,3]. Several conditions, including insulin resistance, hypertension, acute decompensated heart failure, chronic kidney disease (CKD), dyslipidemia, vitamin D deficiency, and metabolic syndrome, are associated with vascular calcification [4,5,6]. Vascular calcification is a predictor of overall mortality and poor arteriovenous graft maturation in patients with CKD [7,8]. As these risk factors can influence the endothelial layer and the smooth muscle cells simultaneously, measures to prevent them are vital.

Recently, mesenchymal stem cells (MSCs) and endothelial progenitor cells (EPCs) were considered important for the development of vascular calcification. MSCs are known as either marrow stromal cells, bone marrow fibroblasts, or skeletal stem cells. They could be classified as bone marrow derived MSCs or pericytes based on their origin [9]. Following their activation by inflammation or specific stimuli, they may differentiate osteogenically, which can activate calcification. Kramann et al. suggested that MSCs within the adventitial layer trigger vascular calcification by translocating into the endothelial and the medial layers [10]. Microvesicles derived from injured endothelial cells induce vascular calcification in part through the attraction of MSCs. Transcriptional modulation by specific agents, such as vitamin D, is a possible therapeutic approach to mitigating such vascular calcification [11]. 

EPCs originating from the bone marrow were shown to be an important mechanism in the repair of injured endothelial cells [12]. However, the EPC phenotype was altered under specific pathologic states, such as the accumulation of uremic toxins [13]. The EPCs with osteogenic character were related to the severity of the vascular calcification [14], and the pharmacologic dose of active vitamin D supplement might enhance the expression of calcifying EPCs in CKD patients [15]. However, nutritional vitamin D supplement may attenuate the severity of vascular calcification or aortic stiffness [16,17]. This significance deserves further clarify. This review explains a possible role of MSCs and EPCs in the mechanisms of vascular calcification and a possible role of vitamin D in that mechanism.

## 2. Mechanism of Vascular Calcification

### 2.1. Endothelial Injury Causing Vascular Calcification

**Figure 1:**
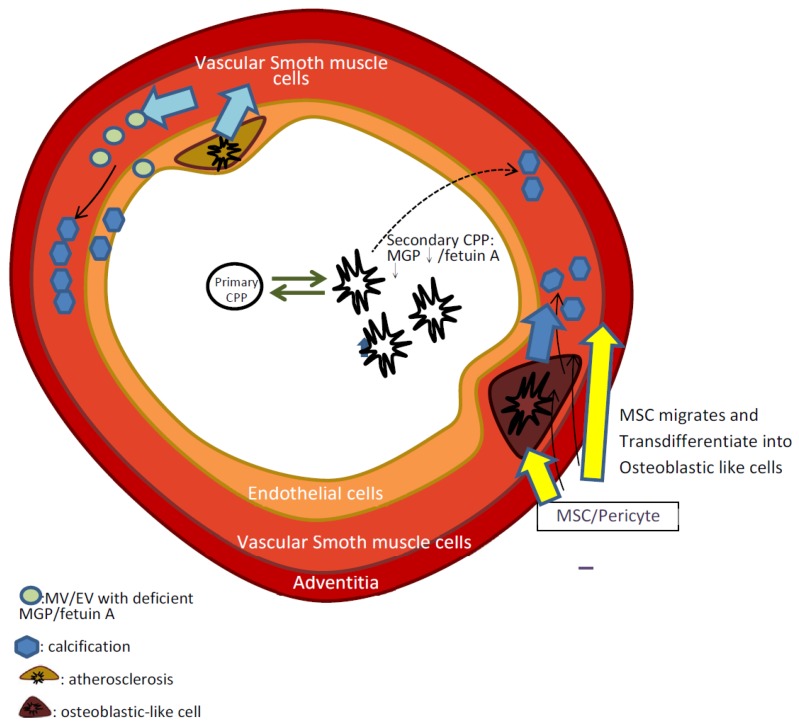
The mechanism of the vascular calcification based on endothelial injury, vascular smooth muscle cell (VSMC) calcification, mesenchymal stem cells (MSCs)/pericytes in the adventitial layer, and the deficiency of calcification inhibitors. Cells from all layers of the vessel wall transformed into osteoblast-like cells. The atherosclerosis within the endothelium induced endothelial calcification by releasing matrix vesicles (MV)/extracellular vesicles (EV) with insufficient matrix Gla protein (MGP)/fetuin A. On the other hand, atherosclerosis also stimulated VSMCs to release MV/EV with insufficient fetuin A after being injured by uremic toxin or renin–angiotensin–aldosterone system (RAAS) activation. The adventitial MSCs/pericyte migrated to the medial layer and transdifferentiated into osteoblast-like cells, which contributed to calcification of the medial layer. CPP: calciprotein particle.

Vascular calcification is characterized by the deposition of hydroxyapatite crystals within the arterial layer, which may originate from atherosclerosis or arteriosclerosis (Figure 1, blue arrow). [1]. Calcifying tissue within the vascular layer may originate from apoptosis within endothelial cells or osteoblastic changes in smooth muscle [18]. The intimal calcification is initiated by the focal retention of apo B–containing lipoproteins in the subendothelial extracellular matrix [19]. In the subendothelial layer, lipid-induced sequential migration of macrophages occurs. The macrophage phagocytizes the lipoprotein cholesterol complex. However, the excessive oxidized lipoprotein induces macrophage apoptosis [18]. The atheroma with apoptotic macrophages and oxidized lipoprotein serves as the necrotic core of the subendothelial layer and initiates the process of mineralization [20]. In addition to subendothelial lipid accumulation, the influences of stress on the endothelial layer, such as the activation of the renin–angiotensin–aldosterone system (RAAS), fluid overload, and insulin resistance, exacerbates the endothelial injury. Montezano et al. demonstrated the direct effect of angiotensin II on endothelial injuries; angiotensin II increased the release of reactive oxygen species by activating vascular nicotinamide adenine dinucleotide phosphate oxidase [21]. Instead of repairing in the endothelial layer, the replacement of the fibrotic tissue by fibroblasts reduced the endothelial compliance. Thus, endothelial injury due to calcification was aggravated by the increased shearing stress. Subendothelial lipid accumulation initiated endothelial injury, and the subsequent inflammation triggered by macrophages and the replacement by hydroxyapatite-associated crystals accelerated atherosclerosis and increased arterial stiffness. 

### 2.2. The Role of Vascular Smooth Muscle Ccells (VSMCs) in Vascular Calcification

Vascular smooth muscle cells (VSMCs) within the medial layer of the arteries underwent rapid morphologic and functional changes after confronting environmental stimuli [22]. Specific stimuli on the smooth muscle layer activated osteoblastic-like differentiation, such as hyperphosphatemia. Hyperphosphatemia is a common complication in CKD patients because of the decrease in the renal clearance of phosphate, which is also related to cardiovascular mortality [23]. Giachelli et al. found that inorganic phosphate promoted the osteogenic differentiation of VSMC directly by induction of a sodium-dependent phosphate transporter (Pit-1) [24]. The core-binding factor α-1 (Cbfa-1) served as the transcription factor activated during the osteogenic differentiation by inducing the expression of tissue-nonspecific alkaline phosphatase [25,26,27]. 

During this osteoblastic transdifferentation process, translation of the Runx-activated canonical Wingless-INT (Wnt)-β-catenin signaling accelerated active calcium deposition and vascular calcification (Figure 1, yellow and blue arrow) [28]. Downstream bone morphogenetic protein 2 (BMP2) was activated by Wnt and propagated the osteogenic differentiation [29]. Hyperphosphatemia also stimulated the serum- and the glucocorticoid-inducible kinase (SGK1) with subsequent activation of the transcription factor NF-kB in VSMCs [30,31]. Therefore, hyperphosphatemia-mediated osteoblastic change within the smooth muscle layer may be a prominent mechanism of vascular calcification. However, the release of matrix vesicles from VSMCs was also associated with vascular calcification [32]. 

In hyperphosphatemia, proliferative VSMCs with low calcitonin and α-smooth muscle actin were found to serve as the transitional form between contractile and calcifying smooth muscle cells. Hyperphosphatemia also stimulated the VSMC apoptosis process [33]. The apoptotic body originating from VSMCs served as the nucleation site of mineral deposition [34]. Inorganic pyrophosphate originated from VSMC serves as the endogenous calcification inhibitors by the ectonucleotide pyrophosphatase/phosphodiesterase (ENPP1) mediated breakdown of nucleotide triphosphates or by the transmembrane protein ankylosis protein homolog (ANKH) mediated transportation. [35]. If the matrix vesicles (MV) contained sufficient calcification inhibitors, such as fetuin-A, the vascular calcification process would be mitigated [33,36]. Therefore, lessening the phosphate burden is important in preventing cardiovascular damage in CKD patients.

Beyond the hyperphosphatemia, protein-bound uremic toxin (e.g., indoxy sulfate) might induce phenotypic changes within the VSMCs by increasing the oxidative stress. Uremic toxins were able to alter the glucose metabolism within the VSMCs (and/or endothelial cells) and therefore increased the cellular release of the calcifying exosome into the artery and worsened the vascular calcification [37]. The uremic toxin also induced the osteoblastic differentiation of VSMCs and promoted further calcification [38]. This evidence indicated that, in CKD patients, the VSMC phenotype might be modulated, and osteoblastic differentiation might be initiated. 

The role of vitamin D on VSMCs has been discussed in many studies. Valcheva et al. noticed that the VSMCs from vitamin D receptor-knock out mice had higher renin activity and premature senescence [39]. Based on the current evidence, in vitro studies demonstrated that vitamin D inhibited the mineralization of VSMCs treated with phosphate and tumor necrosis factor alpha (TNF-α) [40]. On the other hand, Chen et al. provided evidence that 1,25(OH)2D decreased VSMCs treated with endothelin mediated by cyclin-dependent kinase 2 (Cdk-2) activity [41]. Contrary evidence also demonstrated the vitamin D might stimulate vascular calcification by modulating the expression of parathyroid hormone-related peptide or the receptor activator of nuclear factor kappa-Β ligand/osteoprotegerin of VSMC [42,43]. The pharmacologic or the supraphysiologic concentrations of active or nutritional vitamin D might contribute to the vascular calcification in vivo studies [44,45]. Therefore, vitamin D has rather complex effects on calcification from the aspect of VSMC, and more advanced studies are needed to elucidate the role of vitamin D in vascular calcification. 

### 2.3. The Role of Adventitial MSCs and Pericyte in Vascular Calcification

Adventitial MSCs (cluster of differentiation (CD)34+ CD31- CD146- CD45- [46]) are considered to contribute to vascular calcification. From the postmortem study of Yang et al., adventitial calcification occurred during the process of intracranial artery calcification [47], and the measurable adventitial vaso vasorum was predictive of the progressive atherosclerotic change in the intracranial arteries [48]. Researchers demonstrated that MSCs reside within the adventitial layer [46], and that MSC differentiation might be initiated after vascular injury. For instance, angiotensin II sensitized the MSCs with fibrogenic character by activating NF-κB [49]. 

Tang et al. provided evidence that the adventitial MSCs carrying the stem cell antigen 1 (Sca-1) surface protein were activated to repair arterial injuries [50]. At the same time, multiple inflammatory cells lie within the adventitial layer, and the pathologic status might dysregulate the repair process and induce vascular calcification (Figure 1, yellow arrow) [51]. Del Toro et al. reported that, in vivo, the adventitial MSC activated chemokine-mediated monocyte and neutrophil aggregation, thus exacerbating subendothelial injury [52]. Kramann et al. reported that vascular calcification could be reversed after the genetic ablation of glioma-associated oncogene homolog 1 (Gli1) for the migration of MSCs carrying human Gli1 from the adventitial layer to the smooth muscle and the endothelial layers in specific animal models (such as those fed with high-fat diets or nephrectomized rats) [10]. 

Sun et al. demonstrated that human adventitial progenitor cells carrying CD10, a common surface marker of acute lymphoblastic leukemia and lymphoid progenitors, have the potential for osteogenic differentiation through the Sonic hedgehog-signaling pathway [53]. In addition to MSCs, pericytes residing within the adventitia can migrate after intimal injury. Pericytes (with surface markers platelet-derived growth factor receptor (PDGF-R)β, α-smooth muscle actin (αSMA), and Neural/glial antigen 2 (NG-2) [54]) are located within the adventitial layer of the vasa vasorum. Vascular injury induces pericyte differentiation and migration during neointima formation and vascular calcification. After arterial injury, the pericyte itself contributed to the restenosis after arterial injury by modulating the PDGF signaling [54,55]. The role of MSCs within the adventitial layer is still not clear in humans. Based on the recent studies, the adventitial MSCs phenotype could be modified by endothelial injury and arteriosclerosis, and such modifications might worsen the vascular calcification. The strategy on modulating adventitial MSCs could be a new aspect in the future.

### 2.4. The Role of Matrix Vesicles/Exosomes and Calciprotein Particles Containing Insufficient Calcification Inhibitors

Plasma is always supersaturated with respect to the apatitic solid phase [56]. In research on osteoporosis and adynamic bone disease, the exchangeable calcium and phosphate pool was supersaturated, and sequential crystal formation occurred if there were no sufficient calcification inhibitors [57,58]. As mentioned previously, endothelial cells or VSMCs released exosomes or matrix vesicles when damaged. The chemokine homeostasis would be disrupted when recruiting erythrocytes, or platelets could release extracellular vesicles at the damaged endothelium [59]. In osteochondrogenic VSMCs, calcifying cells released matrix vesicles containing calcium, phosphate, lipoprotein, and calcification inhibitors [36]. The released exosome containing specific microRNA(miR), such as miR-135a(*), miR-762, miR-714, and miR-712 [60], or miR-32 [61], could be transported into nearby VSMC in a heparin sulphate proteoglycans (HSPG)-dependent manner [62], and such exosomes could stimulate osteogenic differentiation of VSMC. 

The calcification inhibitors were assembled with apolipoprotein, crystalline, and amorphous hydroxyapatite calcium as calciprotein particles (CPPs) [63]. As the CPPs contained sufficient calcification inhibitors, such as fetuin-A, the CPPs were integrated into spherical rather than unstructured minerals. Such CPPs are called primary CPPs, and the primary CPPs were cleared through the scavenger receptor A, present on hepatic endothelial cells [63,64]. In subjects with insufficient calcification inhibitors, the CPPs turned into unstructured minerals with a diameter of 120–150 nm, which was larger than the primary CPPs (60–70 nm) [6]. These unstructured CPPs are called secondary CPPs, and such secondary CPPs were predictive for vascular calcification and cardiovascular mortality in uremic patients (Figure 1) [65]. Clinical evidence suggested that patients with CKD and a higher concentration of secondary CPPs had a higher incidence of vascular calcification [66]. Therefore, maintaining sufficient calcification inhibitors should be a therapeutic strategy for treating vascular calcification.

Among the calcification inhibitors, matrix Gla protein (MGP) phosphorylation and carboxylation provided the effectiveness for chelating calcium [67]. Vitamin K is essential for the post-translational conversion to γ-carboxyglutamate [68]. Under vitamin K sufficient status, phosphorylated MGP also avoided osteoblastic changes of VSMCs [69]. Mature MGP formed mineralized complex with fetuin-A, calcium, and phosphorus ion to lessen the mineral composition within vessels (Figure 1) [70]. In CKD patients, the secondary CPP was associated with insufficient MGP [71]. It is rational to supply vitamin K in subjects such as CKD patients with vitamin K deficiency [72]. Vitamin D deficiency, which is common in CKD patients, involves a functional vitamin K deficiency [73]. Cashman et al. provided evidence that the vitamin D status was correlated negatively with the uncarboxylated osteocalcin [74]. On the other hand, vitamin D might enhance the carboxylated MGP productions based on in vitro and in vivo evidence. In the osteoblast, vitamin D induced osteogenesis by enhancing ɣ-carboxylated-MGP-containing osteocalcin [75]. After treating the vitamins D and K with the osteoblast from the diabetic mice, the bone anabolism was enhanced [73]. In this manner, the extraosseous calcification might be lessened. Therefore, vitamin D supplements should be another strategy in treating vascular calcification based on the aspects of the CPPs.

## 3. The Role of EPCs, Hematopoietic Progenitor Cells, and MSCs in Vascular Calcification

From the traditional aspects, vascular calcification involves subendothelial hydroxyapatite formation, the osteogenic transformation of smooth muscle cells, and dysregulation/reductions in the activity of calcification inhibitors. In severe cases of ischemic limbs or peripheral occlusive arterial disease, the exhausted production of endothelial/hematopoietic stem cells and bone marrow MSCs contributes to the progression of vascular calcification. 

### 3.1. EPCs and Arterial Calcification

As subendothelial atheroma occludes arteries, hypoxia-inducible factor-1-alpha (HIF-1-alpha) regulates the gene expression of vascular endothelial growth factor (*VEGF*). The activated *VEGF* was shown to modulate matrix metalloproteinase-9 (MMP-9) activity and increase the mobilization of EPCs [76]. In physiological hypoxia, angiogenesis was shown to repair a damaged endothelium by promoting the differentiation of EPCs [77]. The circulating EPCs migrated and invaded the subendothelial region to replace injured endothelial cells and regulated the differentiation of the surrounding stromal cells [78]. However, the circulating EPCs may be stimulated into endothelial regeneration or calcification. For example, in patients with end-stage renal disease, EPCs with surface markers of CD34+/CD133−/KDR+/CD45− were activated by active vitamin D, which lowered the expression of osteocalcin [79]. Furthermore, the concentration of circulating endothelial cells with markers of CD34+/CD133+/KDR+ can predict cardiovascular mortality in patients with atherosclerosis and those requiring hemodialysis [80]. However, EPCs bearing the markers CD34+/CD133+/VEGFR+ can enable vasculogenesis [81]. In patients with CKD, the accumulation of uremic toxin disrupted EPC migration into the endothelium. Wu et al. demonstrated that the protein-bound uremic toxin indoxyl sulfate down-regulated endothelial vacuolization by disrupting the effect of HIF-1-alpha [13]. Thus, indoxyl sulfate disrupted EPCs regeneration and endothelial repair. 

### 3.2. Hematopoietic Progenitor Cells and Arterial Calcification

The hematopoietic progenitor cells originating in the bone marrow can differentiate into the myeloid and the lymphoid progenitor cells under oxidative stress. Dutta et al. first demonstrated in an animal model that a myocardial infarction stimulated hematopoietic progenitor cells production and worsened atherosclerosis [82]. Chronic stress decreased the expression of chemokine (C-X-C motif) ligand 12 CXCL12 within the bone marrow and facilitated the release of inflammatory monocytes and neutrophils [83]. The endothelial chondrocyte-like phenotype is common during vascular calcification, and monocytic cells can be programmed through stimulation of inflammatory cytokines, such as transforming growth factor-1β, to differentiate with chondrocyte characters, such as generate type II collagens [84]. 

Doehring et al. demonstrated that transplanted bone marrow CD34+/CD13+ myeloid progenitor cells transdifferentiated into chondrocyte-like cells in an atherosclerotic animal model [85]. Thus, bone marrow hematopoietic progenitor cells can be conditionally stimulated into monocytes or osteoclasts, which may regulate osteogenesis within the endothelial or the arterial smooth muscle cells. Recently, Cho et al. showed that bone marrow–derived hematopoietic progenitor cells (Sca-1+/PDGFRα−) have osteoclastogenic potency, which can lead to osteoclast-mediated bone resorption. 

As inflammatory cytokines, such as interleukin-1 or interleukin-5, increased, Sca-1+/PDGFRα− decreased and was associated with more severe osteogenesis and vascular calcification within the vascular wall [86]. Recently, Frodermann et al. provided evidence that the exercise decreased the release of hematopoietic progenitor cells from the bone marrow by modulating the leptin release from the adipocyte. In this manner, the cardiovascular damage was relieved by lessening the inflammatory process [87]. This evidence gave us clues that the pathologic status induced the inflammatory differentiation of hematopoietic progenitor cells, and that such inflammation worsened the endothelial injury. Certain interventions lessening the differentiation might be a therapeutic strategy for treating endothelial injuries and sequential vascular calcification. 

### 3.3. MSCs and Arterial Calcification

MSCs are multipotential stromal cells that can differentiate into osteoblasts, chondrocytes, or adipocytes. MSCs reside within adipose tissue, bone marrow, the umbilical cord, and the adventitial/medial layer of the vasculature. Cluster of differentiation (CD) markers indicate the origin of MSCs. For example, stromal stem cells from bone marrow have the surface markers SH2, SH3, CD29, CD44, CD71, CD90, CD106, CD120a, and CD124. The surface markers of MSCs determine whether they have the potential to differentiate into endothelial cells under specific stimuli. Miranville et al. demonstrated that adipose tissue-derived MSCs with CD34+/CD31− markers differentiated into endothelial cells and alleviated neointima formation [88]. However, MSCs residing within tissues other than the adventitial layer contributed to inflammation rather than differentiation into endothelial cells during osteogenic differentiation [89]. This was because MSCs that originated from adipose tissue or bone marrow required collagenase to cleave the hindrance posed by the stromal cells [90]. In summary, adipose MSCs have potential for osteogenic differentiation, and such characteristics might be related to the development of vascular calcification. 

### 3.4. Extracelluar Vesicles and Calciprotein Particles Stimulated by MSCs

Extracellular vesicles are the double-layer phospholipid membrane vesicles released from cells. They encapsulate biological molecules such as nucleic acids, diverse cellular proteins, and metabolites [91,92]. As the extracellular vesicle might contain microRNA or specific proteins, it served as the intercellular communication [91]. MSCs had anti-inflammatory and or immunosuppressive properties [93], and the exosomes released from MSCs were identified as a possible therapeutic target for vascular calcification [94]. G Sahoo et al. showed that the exosome released from human stem cells induced endothelial viability in a paracrine manner [95]. Guo et al. reported that exosomes from bone marrow–derived MSCs bear the surface markers CD63 and CD81. Such exosomes hampered VSMC calcification by modulating the microRNA regulating the mitogen-activated protein kinase (MAPK) or the Wnt signaling pathways [96]. Wei et al. demonstrated that extracellular vesicles isolated from the MSCs and coated with heparin-based vehicles maintained patency after arterial graft in rats. This effect was modulated through the transfection of extracellular vesicles from atherogenic macrophages into anti-inflammatory and antiosteogenic macrophages [97]. From the evidence above, the undifferentiated MSCs had anti-inflammatory and/or immunosuppressive properties, and the extracellular vesicles released from MSCs might be a therapeutic strategy for vascular calcification by reducing inflammation. 

## 4. Possible Therapeutic Roles of Vitamin D in MSCs and Vascular Calcification

Vitamin D is an essential hormone provided through exposure to sunlight or through intake from the diet. There are two major types, ergocalciferol and cholecalciferol. After being radiated by ultra-violet B (UVB) light at wavelengths of 290–315 nm, the ergosterol in plants or fungi is synthesized into ergocalciferol. Cholecalciferol originates from keratinocytes. After being radiated by UVB, 7-dehydrocholesterol is transformed into cholecalciferol [98]. The body’s synthesized cholecalciferol or ingested ergocalciferol/cholecalciferol is transported to the liver by a vitamin D transport protein and hydroxylased within the liver, where the vitamin D is transformed into 25-hydroxy vitamin D (25(OH)D, which is transported to the kidneys to be converted to 1,25(OH)2D by 1-alpha hydroxylase. The 1,25(OH)2D is then transported from the cytoplasm into the nucleus to interact with the vitamin D binding protein, which binds to the vitamin D receptor element so that vitamin D can influence the transcription of specific genes [99]. 

Vitamin D deficiency is common in CKD and diabetes mellitus for several reasons: (1) renal deterioration and proteinuria [100,101,102], (2) reduced 1-alpha hydroxylase activity within the kidney [103,104], (3) increased catabolism of 25(OH)D into inactive metabolite 24,25(OH)2D [103,105], and (4) pharmacological concentrations of vitamin D [106]. Current active vitamin D supplements have microgram concentrations [107]. In vitro studies demonstrated that supraphysiological concentrations of active vitamin D influenced the 25(OH)D production in the liver. 

In CKD patients, 1,25(OH)2D was interfered with by fibroblast growth factor 23 (FGF23). FGF23 is the hormone secreted from osteocytes. In the CKD patients with decreased renal excretion of inorganic phosphates, FGF 23 served as the phosphaturic hormone to decrease the reabsorption of phosphate from the proximal tubule in the kidneys [108]. FGF23 directly suppressed the activity of 1-α hydroxylase and increased the activity of 25-hydroxyvitamin D3-24-hydroxylase [109,110]. The decrease of vitamin D and the increase of FGF23 interfered with the osteogenic differentiation of bone marrow MSCs in CKD patients [111,112,113]. Therefore, the correction of vitamin D deficiency is critical to the treatment of vascular calcification, and the synergy of vitamin D and MSCs should be considered in the treatment of vascular calcification.

Vitamin D deficiency is a risk factor and a predictor for cardiovascular disease [114]. In epidemiological studies, vitamin D deficiency was associated with a higher incidence of hypertension [115], coronary artery disease (CAD) [116], fatal stroke [117], and peripheral arterial disease [118]. Vitamin D deficiency itself was associated with impaired peripheral insulin sensitivity [119] and arterial stiffness [120]. The role of vitamin D in vascular disease involves immune modulation by moderating the release of anti-inflammatory cytokines by macrophages [121] or the reduction of RAAS hyperactivity [122,123]. Moreover, vitamin D can regulate carboxylation of the vitamin K-mediated MGP. Carboxylated MGP chelates excessive calcium and lessens extraosseous calcification. Vitamin D enhances osteocalcin and MGP production within osteoblasts. The downstream carboxylation of osteocalcin and MGP improves bone mineralization and mitigates extraskeletal calcification [6]. Beyond the aspects above, the adjunctive role of the vitamin D on MSCs or EPCs in treating vascular calcification is discussed as below. 

### 4.1. The Influence of Vitamin D on EPCs in Vascular Calcification

Vitamin D receptor expression can predict cardiovascular disease. Ai et al. demonstrated that patients with CAD had fewer vitamin D receptors on EPCs than did control patients (Table 1) [124]. Vitamin D supplementation can accelerate EPC migration and differentiation through an angiogenesis-associated pathway. Grundmann et al. showed that endothelial colony-forming cells expressed mRNA of VEGF and pro-matrix metalloproteinase (pro-MMP) activity after treatment with physiological concentrations of 1,25(OH)2D in vitro (Table 1) [125]. Additionally, Schröder-Heurich et al. demonstrated that 1,25(OH)2D increased endothelial progenitor adhesion by alleviating the inflammatory signals of TNF-α in vitro (Table 1) [126]. 

Yu et al. found, in vitro, that physiological concentrations of 1,25(OH)2D altered the RNA expression profile of EPCs treated with high glucose [127]. Differentially expressed RNA influenced the activity of MMP and guanosine-5’-triphosphatase, which are related to EPC migration. These in vitro studies demonstrated that 1,25(OD)2D supplementation at physiological concentrations improved the adhesion of the EPCs in the injured endothelium and stimulated the migration of EPCs from the bone marrow. 

Schröder-Heurich et al. also demonstrated that the adequate vitamin D supplement promoted the formation of VE-cadherin adhesion junctions on the EPCs. In this manner, the endothelial barrier integrity pretreated with TNF-α was repaired. Xu et al. also demonstrated that, in vitro, vitamin D alleviated EPC injuries, which were treated with Ang II by modulating the PPAR-γ/HO-1 pathway. The angiogenesis impaired by Ang II would be restored after vitamin D was supplied at cellular level (Table 1) [128]. At the same time, the study from Hammer et al. provided evidence that the calcitriol supplement improved EPCs viability in vitro (Table 1) [129]. These in vitro studies showed the possible therapeutic effect of the vitamin D on EPC migration and adhesion as well as the enhancement of the endothelial integrities under the circumstances involving vascular injury. 

### 4.2. The Role of Vitamin D and MSCs/Pericytes in Vascular Calcification

**Figure 2:**
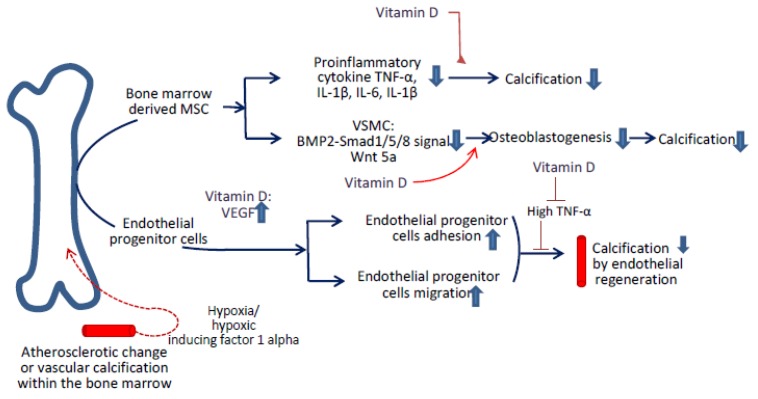
The adjunctive role of vitamin D in treating vascular calcification based on MSCs and endothelial progenitor cells (EPCs). In physiological hypoxia, angiogenesis can repair a damaged endothelium by promoting the differentiation of EPCs. The circulating EPCs migrated and invaded the subendothelial region to replace the injured endothelial cells; they also regulated the differentiation of the surrounding stromal cells and therefore reduced calcification. The MSCs mitigated calcification by lowering the proinflammatory cytokines or reducing the VSMC osteogenic expression. Vitamin D served as the adjunctive role in mitigating calcification by influencing EPCs and MSCs in several manners. For EPCs, vitamin D enhanced the EPCs mobilization during angiogenesis by increasing the vascular endothelial growth factor (VEGF) release. Vitamin D also enhanced the EPC adhesion and migration. Under the inflammatory status, such as high TNF-α scenario, the further vascular calcifications could be lessened by the usage of vitamin-D. For MSCs, vitamin D influenced the secretion of proinflammatory cytokines, such as TNF-α, interleukin 1(IL-1), and interleukin 6 (IL-6), which might induce osteogenic MSCs. Vitamin D decreased the VSMC osteogenic differentiation by decreasing the BMP2-Smad1/5/8 (mothers against decapentaplegic homolog 1/5/8) signal or Wnt5a expression.

Beyond the ability to differentiate osteoblasts, adipocytes, and chondroblasts, MSCs demonstrated anti-inflammatory and immune regulation functions [130,131]. An in vivo study initiated by Kramann et al. showed that the osteoblast-like character was initiated under specific circumstances, such as uremia [132]. The inflammatory cytokines released from the injured aorta, such as TGF-*β*1, mobilized MSC migration for neointimal formation [133]. However, Wang et al. provided in vitro evidence that the conditioned medium from MSCs retarded the VSMC osteoblastic change by blocking the bone morphogenetic protein (BMP) signaling and decreasing inflammatory cytokines in vitro [134,135]. Based on the in vitro evidence above, MSCs might provide the protective role in a paracrine manner to influence the calcification process, including anti-inflammatory effects, blocking the BMP2-Smad1/5/8 signal, downregulating the Wnt signal within VSMC, or attenuating the apoptosis of VSMC (Figure 2) [134,135,136,137].

Vitamin D deficiency was related to adventitial inflammation in clinical studies. Oma et al. noticed that the vitamin D concentration was inversely correlated with the monocyte infiltration within the adventitial layer in patients with CAD and inflammatory rheumatic disease [138]. Additionally, the vitamin D associated gene expression within aortic tissue might be influenced in patients with rheumatoid arthritis. Paraoxonases 2, which had antioxidative properties during atherosclerotic processes, was regulated by vitamin D. The expression was lessened during the inflammation [139]. Vitamin D was associated with lessening the inflammatory cytokine. 

From the in vitro study initiated by Wang et al., the culture medium of MSCs decreased the calcium deposition in the VSMC because of the decreased expression of TNF-α, IL-1β, and IL-6 (Figure 2) [134]. Wasniks et al. also noticed that vitamin D decreased the TNF-α, and IL-6 secretions within osteocytes by suppressing M1 macrophages and influencing the osteogenic expression of MSCs [140]. Vitamin D had several roles in reducing the IL-1β-stimulated inflammatory profile in the adipocyte tissue, and such characteristics might be applied in lessening the calcification in MSCs in vitro [141]. 

A low vitamin D diet was observed to induce vascular calcification through the activation of BMP2 within the VSMC [142]. Fu et al. found that 1,25(OH)2D suppressed BMP2 activity in the bone marrow MSCs by binding the BMP2 promoting region [143]. Goltzman et al. also provided in vivo evidence that the vitamin D that originated from the osteocyte directly decreased the BMP2 release into serum and then mitigated the extraskeletal calcification [144]. 

Human marrow-derived MSCs (marrow stromal cells, hMSCs) give rise to osteoblasts, and their differentiation is stimulated by 1α,25(OH)2D, although hMSCs can also synthesize 1α,25(OH)2D. CKD reduces 1α,25(OH)2D production in kidneys and human MSCs [112]. Indeed, the vitamin D metabolism in hMSCs is regulated, as it is in the kidneys, and this promotes osteoblastogenesis in an autocrine/paracrine manner. CKD is associated with elevated circulating fibroblast growth factor 23 (FGF23). In vitro, rhFGF23 counters vitamin D-stimulated osteoblast differentiation of hMSCs by reducing the vitamin D receptor, CYP27B1/1α-hydroxylase, biosynthesis of 1α,25(OH)2D3, and signaling through BMP-7. Thus, the dysregulated vitamin D metabolism in hMSCs may contribute to impaired osteoblastogenesis and altered mineral metabolism in CKD subjects [113].

MSCs have the ability to reduce the VSMC calcification through down-regulating the Wnt signaling pathways. Guan et al. found that the culture medium from MSCs decreased the VSMC osteogenic differentiation by lowering Wnt 5a (Figure 2) [145]. Vitamin D regulated the expression of Wnt 5a in other systems, such as the respiratory tract [146]; therefore, it might provide a conjunctive role in decreasing vascular calcification. 

From the evidence mentioned above, the adventitial MSCs carrying Gli-1 differentiated into osteoblast-like cells in the medial layer. However, the role of vitamin D on the MSCs in the adventitial layer is still under investigation. Recently, Hegner et al. noticed that the expression of the mammalian target of rapamycin (mTOR) influenced the calcification of MSCs in vitro [147]. They found that the activation of mammalian target of rapamycin complex 1 (mTORC1) was associated with the calcification of MSCs. When inhibiting mTORC1 by rapamycin, the mammalian target of rapamycin complex 2 (mTORC2) activity increased with a lessening of the calcification in MSCs. Vitamin D inhibited the mTORC1 activity through the inhibition of the tuberous sclerosis protein complex [148]. From this aspect, vitamin D might modulate the MSCs within the adventitia directly or influence the microenvironment.

### 4.3. The Role of Vitamin D in Adipose Tissue-Derived Stem Cells

The previous sections revealed that adipose tissue-derived MSCs have multipotency for differentiation into chondrocytes or smooth muscle cells. Adipose tissue-derived MSCs have vitamin D receptors within the nucleus, and the supplementation of the active form of vitamin D stimulated CYP24A1 activity and reduced 1,25(OH)2D expression within MSCs. However, the supplementation of the 25(OH)D increased intracellular active vitamin D production [149]. Thus, adipose tissue-derived MSCs can be modulated by vitamin D, especially nutritional vitamin D (e.g., cholecalciferol). From the study of Pesarini et al., vitamin D decreased the viability in time- and dose-dependent manners on the adipose tissue-derived MSCs and decreased the further adipose tissue formation [150]. 

Vitamin D induced the adipocyte stem cell osteogenic changes through activating bone morphogenetic protein 2 (BMP2) signaling [151]. At the same time, the supplementation of vitamin D modulated the chemokine-mediated inflammation induced by adipose tissue [152]. The vitamin D supplement might modulate the miR expression in the adipose tissue. Karkeni et al. also provided evidence that vitamin D lowered NF-κB signaling by alleviating the expression of miR 146a and miR-150 [153]. Thus, vitamin D decreased the adipocyte formation from stem cells by inducing apoptosis and modulating the inflammatory cytokine release within the adipocyte. 

In addition to MSC migration, vitamin D may influence the differentiation of MSCs into adipocytes. MSCs within the bone marrow are the molecular switch between the osteoblastogenic and the adipocytic transformation. Several pathways, such as C/EBP-γ, C/EBP-α, and peroxisome proliferator-activated receptor-γ pathways, regulate MSC differentiation [154]. Vitamin D contributes to bone formation by activating the Wnt/β-catenin pathway. Lu et al. showed that active vitamin D induced bone formation by increasing the secretion of Wnt 10b by osteoclasts [155]. Therefore, vitamin D may play an adjunctive role in alleviating adipocyte transformation in MSCs and reducing the inflammation associated with vascular calcification. 

The aforementioned evidence reveals that vitamin D may play a substantial role in modulating the therapeutic effect of MSCs in the treatment of vascular calcification. 

## 5. Conclusions

Vascular calcification involves the deposition of calcifying particles within the endothelial and the medial layers after vascular damage. Recent reports on the MSCs lying within the adventitial layer demonstrated their role in developing vascular calcification. Therefore, the possible role of progenitor cells originating from bone marrow and soft tissue should be emphasized. Vitamin D deficiency is an important factor contributing to vascular calcification. Supplementation of vitamin D might modulate the calcification by modulating the MGP carboxylation. On the other hand, vitamin D might influence the phenotype of EPCs, hematopoietic progenitor cells, and MSCs. Vitamin D may be targeted along with MSCs in the treatment of vascular calcification. 

## Figures and Tables

**Table 1:** The influence of vitamin D on EPCs in the development of vascular calcification.

Performance of EPCs	Characteristics	Surface Marker
Vitamin D receptors on EPCs	Decrease in coronary artery disease (CAD) [124]	CD45dim, CD34+, and KDR+
EPCs migration and differentiation	Accelerated [125]	CD34+, CD31+, CD45−, and CD133−
Endothelial colony-forming cells expressed mRNA of VEGF and pro–matrix metalloproteinase (pro-MMP) activity	Increased [125]	CD34+, CD31+, CD45−, and CD133−
Endothelial progenitor adhesion	Increased [126]	CD31+, CD45+, and CD133+
Migration of the EPCs from the bone marrow	Increased [127]	1,1′-Dioctadecyl-3,3,3′,3′-tetramethylindocarbocyanine-labeled acetylated low density lipoprotein and fluorescein isothiocyanate -Ulex europaeus agglutinin-1
Formation of VE-cadherin adhesion junctions on the EPCs	Increased [126]	CD31+, CD45+, and CD133+
EPC injury by Ang II through modulating the PPAR-γ/HO-1 pathway	Decreased [128]	VEGF-2+ and CD13+
EPC viability	Improved [129]	CD34+ and KDR+

## Abbreviation

αSMA	α-smooth muscle actin
Ang	angiotensin
BMP	bone morphogenetic protein
BMP2	bone morphogenetic protein2
*C*/*EBP*	CCAAT/enhancer binding protein
CAD	coronary artery disease
CBFA1	core-binding factor α-1
CD	cluster of differentiation
Cdk-2	Cyclin-dependent kinase 2
CKD	chronic kidney disease
CXCL1	Chemokine (C-X-C motif) ligand 1
CXCL12	Chemokine (C-X-C motif) ligand 12
CYP24A1	Cytochrome P450 family 24 subfamily A member 1
EPCs	endothelial progenitor cells
FGF23	Fibroblast growth factor 23
Gli1	The Human Glioma-Associated Oncogene Homolog 1
HIF-1-alpha	hypoxia-inducible factor-1-alpha
IL-1	interleukin 1
IL-6	Interleukin-6
KDR	kinase insert domain receptor
LDL	low-density lipoprotein
M1 macrophage	classically activated macrophage
MAPK	mitogen-activated protein kinase
MGP	matrix Gla protein
miR	MicroRNA
MMP	matrix metalloproteinase
MSCs	mesenchymal stem cells
mTOR	mammalian target of rapamycin
mTORC1	mechanistic target of Rapamycin complex 1
mTORC2	mechanistic target of Rapamycin complex 2
MV	Matrix vesicle
NF-kB	nuclear factor kappa-light-chain-enhancer of activated B
NG-2	Neural/glial antigen 2
PDGFR	Platelet-derived growth factor receptors
PDGFRβ	Platelet-derived growth factor receptor beta
PPAR-ɣ	peroxisome proliferator-activated receptor gamma
RAAS	renin-angiotensin-aldosterone system
Sca-1	stem cell antigen 1
SGK-1	serum- and glucocorticoid-inducible kinase 1
Smad 1/5/8	Mothers against decapentaplegic homolog 1/5/8
TNF-α	tumor necrosis factor alpha
UVB	ultraviolet B
VEGF	vascular endothelial growth factor
VSMC	vascular smooth muscle cell
Wnt	Wingless-INT
